# Adolescent predictors of objectively measured physical activity and sedentary behaviour at age 42: the Amsterdam Growth and Health Longitudinal Study (AGAHLS)

**DOI:** 10.1186/1479-5868-8-107

**Published:** 2011-10-02

**Authors:** Léonie Uijtdewilligen, Amika S Singh, Jos WR Twisk, Lando LJ Koppes, Willem van Mechelen, Mai JM Chinapaw

**Affiliations:** 1 Department of Public and Occupational Health, EMGO+ Institute for Health and Care Research, VU University Medical Center, Amsterdam, The Netherlands; 2 Department of Health Sciences, Section Methodology and Applied Biostatistics, VU University Medical Center, Amsterdam, The Netherlands; 3 Department of Epidemiology and Biostatistics, VU University Medical Center, Amsterdam, The Netherlands; 4 Division Work and Employment, TNO, Hoofddorp, The Netherlands; 5 Body@Work, Research Center Physical Activity, Work and Health, TNO-VU University Medical Center, Amsterdam, The Netherlands

**Keywords:** Accelerometry, Aerobic fitness, Longitudinal, Motor fitness, Personality

## Abstract

**Background:**

This study investigated the associations of physical characteristics and personality in adolescence with physical activity and sedentary behaviour in adulthood.

**Findings:**

Physical characteristics (i.e. objectively measured BMI, sum of skin folds, MOPER test battery performance), and personality (i.e. self-reported inadequacy, social inadequacy, rigidity, self-sufficiency/recalcitrance, dominance, achievement motivation, facilitating anxiety, debilitating anxiety, and social desirability) were assessed in 217 adolescent boys (Mean 13.0, SD 0.6) and girls (Mean 12.9, SD 0.6). Twenty-nine years later, at the age of 42, their physical activity and sedentary behaviour were assessed by means of accelerometry. Boys who scored lower on self-sufficiency/recalcitrance and higher on facilitating anxiety spent more time sedentary in adulthood. Girls with a superior standing high jump performance, and a lower score on social desirability spent more time sedentary in adulthood. In contrast with sedentary behaviour, physical activity at age 42 year could not be predicted by physical characteristics or personality in adolescence.

**Conclusions:**

Sedentary behaviour in adulthood was partly explained by physical characteristics and/or personality in adolescence. Thus, our results suggest that it may be possible to identify people who are at risk of becoming sedentary at a rather young age.

## 1. Introduction

Physical activity and sedentary behaviour are generally accepted as being two distinct classes of behaviour, which have been shown to be independently associated with energy expenditure, body weight, and metabolic factors [1-3].

Although a substantial body of literature has focused on drivers for adopting an inactive lifestyle the majority of these studies are cross-sectional [4]. The association between age, education, self-efficacy and physical activity has been frequently investigated, whereas other factors such as personality and fitness have been rarely examined [4]. Research on sedentary behaviour is rapidly growing [5,6], however, evidence on its determinants is relatively scarce.

Considering the above, research investigating under reported determinants of physical activity and sedentary behaviour in a prospective design and by means of objective measurement instruments is of great importance [4-6]. Therefore, the present study aims to extend existing knowledge by investigating which physical characteristics and personality in adolescence are longitudinally associated with objectively measured physical activity and sedentary behaviour in adulthood.

## 2. Methods

### 2.1 Participants and procedures

We used data collected at wave 1 (1976/77) and wave 10 (2006) of the Amsterdam Growth and Health Longitudinal Study (AGAHLS). This longitudinal study started in 1976/1977 monitoring growth, health, and lifestyle in more than 600 boys and girls aged 13 years. The study rationale, recruitment procedures and protocol have been reported in detail elsewhere [7]. We included participants with physical characteristics and/or personality data at wave 1 and data on physical activity and/or sedentary time at wave 10, resulting in a sample of 217 participants (33% of the baseline sample; 42% male). Compared to those with complete data, participants without wave 10 data had a significant higher BMI, performed better in the arm pull test and scored higher on social desirability at baseline. The AGAHLS was approved by the medical ethics committee of the VU University Medical Center, Amsterdam, The Netherlands. All subjects gave their written informed consent [7].

### 2.2 Measurements in adolescence

#### 2.2.1 Physical characteristics

Body height and weight were measured using a Harpenden digital readout, wall-mounted or portable stadiometer (Holtain, UK), and a spring balance (Van Vucht, the Netherlands), and BMI (kg/m^2^) was calculated. The sum of four skin folds (biceps, triceps, subscapular and supraliliac) was used as indicator of body fatness and measured with a Harpenden calliper (Holtain, UK) [8].

Aerobic fitness was assessed by measuring the maximal oxygen uptake (VO_2_max) while running on a treadmill (Quinton 18-45, USA). During the entire run, the expired air was analysed on O_2 _and CO_2 _by the Ergoanalyzer (Jaeger, the Netherlands), and subsequently expressed in VO_2_max (ml·min·kg^-2/3^) relative to the individuals' body weight [9].

Muscular fitness, i.e. the respondents' strength, speed, flexibility and endurance capacity was measured by means of the MOPER test battery including 8 different tests. The MOPER components are described in table 1[10]. Validity and reliability of the MOPER tests have been shown to be acceptable in children [11,12].

**Table 1:** Description of the 8 MOPER elements

MOPER test	Description
*Strength*	
1. Arm pull	The maximal force (in kg) pulled with the preferred arm while standing
2. Standing high jump	The maximal standing vertical jump height (in cm)
3. Flexed arm hang	Maximal time (in sec) that eyes were kept above a horizontal bar hanging in a bent arm position
4. 10 leg lifts	Time (in sec) needed for lifting the legs 10 times from horizontal to vertical position with stretched knees while lying
*Speed*	
5. Sprinting	Time (in sec) needed to run 10 times between two lines which were 5 meters apart
6. Plate tapping	Time (in sec) needed to tap 50 times with 'best' hand between two plates which were 75 centimetres apart
*Flexibility*	
7. Sit-and-reach	Maximal reach (in cm) while sitting with extended knees
*Endurance*	
8. Endurance run	Maximal distance (in km) covered in 12 minutes

#### 2.2.2 Personality

Personality traits were assessed using the youth versions of the Dutch Personality Inventory (DPI) [13], and the Achievement Motivation Test (AMT) [14]. The DPI assessed the participant's inadequacy, social inadequacy, rigidity, self-sufficiency/recalcitrance, and dominance. The AMT assessed the participants' achievement motivation, facilitating anxiety, debilitating anxiety, and social desirability. Psychometric properties of the DPI and AMT are presented in table 2.

**Table 2:** Psychometric properties of the personality constructs based on figures of Luteijn et al. [13] and Hermans [14]

Construct	# items	Scoring	Reliability	Validity
*DPI^a^*			*Chronbachs alpha^b^*	*Correlations with school/parent report*
Inadequacy (e.g. having vague physical complaints)	28	Example question: I do not make friends easily	.85; .87	-.20 (cognitive functioning) -.25 (concentration, ability to work on and work independently)
Social inadequacy (e.g. avoiding social contacts)	13	Scale: true (scored 2), not true (scored 0), ? (scored 1) for all questions	.75; .82	.26 (behavioural assessment)
Rigidity (e.g. the need for regularity)	25	Sum score: the higher the more	.76; .83	.26 (cognitive functioning) .22 (achievement motivation)
Self-sufficiency/recalcitrance (e.g. mistrust of others)	24		.74; .75	-.23 (cognitive functioning) -.27 (social-motivational functioning)
Dominance (e.g. trying to be the boss)	15		.59; .70	.19 (parental perception of child)
*AMT^c^*			*Test-retest correlations^d^*	*Correlations with grades^e^*
Achievement motivation (e.g. the need to achieve)	39	Example question: I feel sometimes/seldom/never bored	.48; .74	.18; .35
Facilitating anxiety (fear of failure, leading to higher achievements)	17	Scale: all questions have different answering options on a three or four point scale	.46; .68	.05; .17
Debilitating anxiety (fear of failure, leading to lower achievements)	15	Sum score: the higher the more	.47; .72	-.17; -.25
Social desirability (e.g. the tendency to give the most socially acceptable answers)	23		.40; .81	.01; .07

^a ^DPI = Dutch Personality Inventory

^b ^Numbers represent a range of Chronbachs alpha among different experimental groups (i.e. primary school pupils, secondary school pupils and 'general')

^c ^AMT = Achievement Motivation Test

^d ^Numbers represent a range of test-retest correlations among boys and girls in different age groups

^e ^Numbers represent a range of correlations between the ATM constructs and grades during different periods of the curriculum (i.e. Christmas and grade transition)

### 2.3 Objectively measured physical activity levels and sedentary time in adulthood

Physical activity was objectively measured using ActiGraph accelerometers (Model GT1M, ActiGraph, LLC, Fort Walton Beach, FL). At age 42, 345 participants were instructed to wear an accelerometer attached to a provided waist belt, for eight consecutive days during waking hours but not during water activities. The accelerometers were set to record acceleration and movement frequency at 60-second epochs. Data were considered eligible for analyses if the respondent had worn the accelerometer for at least one day for ≥ 500 minutes per day. From the accelerometer data we computed two scores: physical activity (counts/min), and time spent sedentary (min/day) [15].

In total, 104 participants (30%) did not provide ActiGraph data. Subjects with and without ActiGraph data were reasonably equal in terms of self-reported physical activity and sedentary behaviour. Of the remaining 241 participants, 12 (5%) wore the accelerometer for < 500 minutes per day and were thus excluded from analyses. Those participants recorded significantly less counts per minute, less sedentary time and less wearing days.

### 2.4 Statistical analyses

We conducted all analyses for males and females separately. We used multiple regression analyses to investigate the associations of physical characteristics and personality in adolescence with physical activity (counts/min) and sedentary behaviour (min/day) in adulthood. We entered all physical characteristics in one block while correcting for skeletal maturation, and removed variables with the lowest p-value from the model until only variables with a p-value < .05 remained. The same was done for personality, though we did not correct for skeletal maturation in these analyses. For all analyses we used the Statistical Package of Social Sciences, 15.0 for Windows (SPSS inc., Chicago, Illinois, USA).

## 3. Results

Table 3 presents descriptive data of the participants during adolescence and adulthood.

**Table 3:** Descriptive data of the male and female participants in adolescence and adulthood

	Males (N = 92)		Females (N = 125)	
	Mean	**S.D**.	Mean	**S.D**.
*Adolescence*				
Age (y)	13.0	0.6	12.9	0.6
Height (cm)	157.9	7.7	159.8	7.8
Weight (kg)	41.8	6.4	45.4	7.5
BMI (kg/m^2^)	16.9	1.4	17.7	2.1
Sum of four skin folds (cm)	2.7	0.9	3.6	1.3
				
*Adulthood*				
Physical activity (counts/min)^a^	344.3	109.6	349.9	99.8
Sedentary time (min/day)^a^	517.7	89.5	457.8	70.4
Wear time accelerometer (days)	7.9	2.1	8.0	1.7

^a ^To be included in the analyses, participants had to wear the ActiGraph for at least one day, for ≥ 500 minutes

Multivariate regression analyses revealed no significant associations between physical characteristics and/or personality in adolescence and physical activity in adulthood (data not shown). Regarding sedentariness, in males, a lower score on self-sufficient/recalcitrant and a higher score on facilitating anxiety was associated with more minutes spent sedentary in adulthood. In females, a superior standing high jump performance, and a lower score on social desirability were associated with more minutes spent sedentary at age 42 (Table 4).

**Table 4:** Prediction model of sedentary time (min/day) at the age of 42 years for males and females

Model		Constant	β	CI	p-value	R^2a^
*Males*						
1^b^	Self-sufficiency/recalcitrance	639.01	-3.92	-6.82; -1.01	.01	36.3
	Facilitating anxiety		5.13	.08; 10.19	< .05	
*Females*						
1^b^	Social desirability	479.24	-4.35	-8.59; -.12	.04	4.3
2^c^	Standing high jump	376.55	2.82	.26; 5.39	.03	3.9

^a ^Values of R^2 ^are multiplied by 100, numbers represent percentages

^b ^Model 1 included all personality characteristics entered in one block

^c ^Model 2 included all physical characteristics entered in one block

## 4. Discussion

Previous studies suggest that people with an 'easy going' personality practice healthier lifestyles [16,17]. However, we found that male subjects who possessed more self-sufficiency/recalcitrance were less sedentary as adults. Individuals with a more self-sufficient/recalcitrant personality, characterised by higher levels of rebellion and hostility [13], might be more restless and volatile and thus engage in less sedentary behaviour.

Males who scored higher on facilitating anxiety, characterised by impulsivity and sensation/stimulation seeking, spent more time being sedentary in adulthood. Also, a superior standing high jump performance in girls was associated with more sedentary time in adulthood. Previous studies found that similar personality and physical characteristics were positively associated with physical activity; i.e. extravert and conscientious people were more physically active[18], and sufficient levels of muscular fitness were predictors of physical activity at a later age [19,20]. In our study these characteristics predicted sedentary time as well. This supports the assumption that physical activity and sedentary behaviour are two different types of behaviour [1-3], and that people who are sufficiently physically active can be highly sedentary at the same time. Since little evidence on determinants of sedentary behaviour is available, more prospective research needs to be conducted to confirm our findings and establish the mechanisms causing these relationships.

To the best of our knowledge, up to now the association between social desirability and sedentary time has only been explored by Jago and colleagues [21]. Although Jago and colleagues examined a slightly different study sample (10 to 14-year old Boy Scouts) with different measures (self-reported sedentary time instead of accelerometry) using a cross-sectional design, a comparable inverse association between social desirability and sedentariness was found. A possible explanation for this association might be that people with a less social desirable nature care less about prevailing norms in society and therefore participate less in social desirable behaviour. Currently much attention is paid to initiatives trying to increase people's physical activity level and decrease their time spent sedentary [22]. People who score low on social desirability may be less likely to participate in such initiatives.

## Limitations

Several limitations are noteworthy. First, participants were rather active as compared to the general Dutch population [23], which may be explained by the participants' relatively high educational background [24]. Therefore, the current results may not be generalisable to the Dutch population. Second, our study sample significantly differed from the baseline sample and from subjects who did not provide ActiGraph data at age 42 which may have biased our results Third, accelerometry is not a gold standard for measuring physical activity nor sedentary time. Although accelerometry provides real time data storage, it does not provide qualitative information on the type of activity. Besides, accelerometry underestimates some activities, such as cycling. Since cycling is a common method of transportation in the Netherlands, underestimation of physical activity may have occurred. Therefore, our findings should be interpreted with caution.

## Conclusion

Sedentary behaviour in adulthood was partly explained by physical characteristics and/or personality in adolescence. Our findings need to be confirmed in other studies.

## Competing interests

The authors declare that they have no competing interests.

## Authors' contributions

LU performed the statistical analyses, interpreted the data and drafted the manuscript. AS participated in the design of the study, contributed to the analyses and interpretation of data and provided critical revision of the manuscript. JT participated in the fund raising, conception and design of the current study, provided statistical expertise and critical revision of the manuscript, and participated in the conception, design and data acquisition of AGAHLS. LK participated in the fund raising, conception and design of the study, and provided critical revision of the manuscript. WM provided critical revision of the manuscript, and participated in the conception, design, fund raising and data acquisition of AGAHLS. MC participated in the design of the study, contributed to the analyses and interpretation of data and provided critical revision of the manuscript. All authors read and approved the final manuscript.
